# Compensability index for compensation radiotherapy after treatment interruptions

**DOI:** 10.1186/1748-717X-7-208

**Published:** 2012-12-08

**Authors:** Paul Martin Putora, Michael Schmuecking, Daniel Aebersold, Ludwig Plasswilm

**Affiliations:** 1 Department of Radiation Oncology, Kantonsspital St. Gallen, Rorschacherstrasse 95, St. Gallen, 9000, Switzerland; 2 Department of Radiation Oncology, Inselspital, Bern University Hospital, University of Bern, Freiburgstrasse, Bern, 3010, Switzerland

**Keywords:** Radiotherapy, Radiation, Interruption, Treatment, Brake, Compensation, Compensability index

## Abstract

**Background:**

The goal of our work was to develop a simple method to evaluate a compensation treatment after unplanned treatment interruptions with respect to their tumour- and normal tissue effect.

**Methods:**

We developed a software tool in java programming language based on existing recommendations to compensate for treatment interruptions. In order to express and visualize the deviations from the originally planned tumour and normal tissue effects we defined the compensability index.

**Results:**

The compensability index represents an evaluation of the suitability of compensatory radiotherapy in a single number based on the number of days used for compensation and the preference of preserving the originally planned tumour effect or not exceeding the originally planned normal tissue effect. An automated tool provides a method for quick evaluation of compensation treatments.

**Conclusions:**

The compensability index calculation may serve as a decision support system based on existing and established recommendations.

## Background

Radiation oncology plays a central role in the treatment of many cancers [1]. The basis of this treatment lies in the different susceptibility and repair capacity of tumour and normal tissues [2]. Radiobiological factors influencing the resistance of cancers to radiotherapy treatment include among others: repopulation, re-oxygenation, repair of sub--lethal damage and re-assortment of cells within the cell cycle [3].

Although several models to estimate and calculate the effect of radiotherapy in cancer as well as normal tissues, including acute and late effects, have been proposed, the alpha-beta model has found widest acceptance in clinical routine [4-6]. Even though most models do not account for the full complexity of interactions as they are known today, available practical models rely on the alpha-beta model and may include a factor that is to account for repopulation.

The estimates of repopulation as well as alpha/beta values differ between tumour types and any prediction derived from these results is less precise than the data they are based on [7,8].

A practical procedure has been proposed by the Royal College of Radiologists. This was chosen as a basis for our calculations. Within their recommendation the repopulation in head and neck cancer is compensated for by subtracting 0.9Gy per treatment day starting after the 4th week [9]. Of course this is a simplified model and cannot account for every inter- and intra- tumour variability, feasible ranges are most likely from 0.5-0.9Gy [10]. Of course the same uncertainty applies to the 4^th^ week [11,12]. The values are very much dependent on the tumour histology.

Treatment breaks due to technical problems, organisational or patient-related issues, holidays or even side effects of treatment may arise. These treatment interruptions are usually not accounted for sufficiently when treatment is simply continued with the originally planned fractionation. If a sub-optimal compensatory regime is chosen the tumour control rate may be jeopardized or unacceptable side effects risked.

Radiation oncologists are often confronted with the decision to adapt a radiotherapy regime when unscheduled interruptions take place. The decision may be to compensate by additional treatment fractions, adapting the dose per fraction of the remaining fractions or changing both, or even the timing (e.g. twice daily treatments). Depending on the clinical scenario, not adapting a radiotherapy regime and accepting deviations may also be an option.

Increasing the total dose (by increasing the number of fractions or increasing the dose per fraction) to adapt to the same expected tumour control rate may not always be the best solution.Many treatment regimens are determined and limited by the possible acute or late side effects. By increasing the dose to compensate for treatment brakes one might increase the risk for side effects on normal tissues.

When established regimes are changed, the available data guiding our decisions becomes thinner. This is why a solid justification is required for tampering with established radiotherapy regimes.

When a compensatory treatment is calculated to correct for the effect on tumour tissue, it is necessary to recalculate the effect on normal tissues as well [9]. The clinician is then faced with two factors in his decision: the new tumour biologically effective dose (ideally approaching the originally planned effect against the tumour) and the biologically effective dose to organs at risk (representing the risk for side effects), especially the late side effects probability. Ideally the compensating treatment should not increase the risk for normal tissue complications.

## Materials and methods

For a given number of days to compensate there are two extremes:

### Offensive compensation

The compensating treatment is defined in such a way as to achieve the same effect in the tumor tissue: i.e. 100% of the original dose.

### Defensive compensation

The other extreme is defining the compensatory treatment in such a way as to avoid increasing risk to normal tissues, i.e. the late normal tissues complication probability remains the same as in the initially planned treatment. This might be of interest in plans that are designed based on the dose constraints of critical organs, such as the brainstem or optic chiasm for example.

The aggressivity of a compensated treatment plan will be judged differently in palliative or curative intention. One always needs to keep in mind that there are limitations in the values used, which are based on estimates.

### Aggressivity

**Figure 1:**
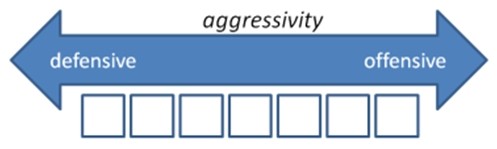
Aggressivity ranges from defensive to offensive compensation.

We term the axis from defensive to offensive compensation *aggressivity* (see Figure 1).

**Figure 2:**
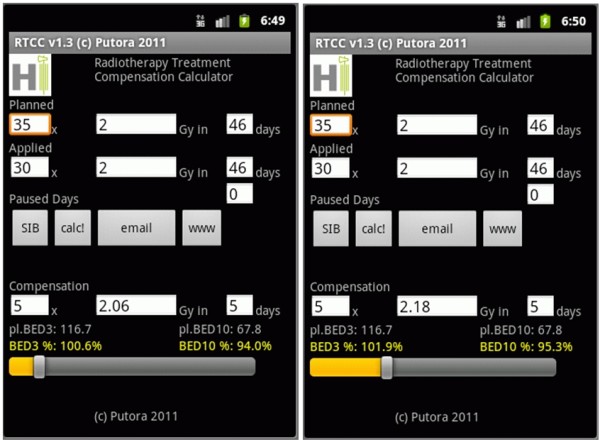
A radiotherapy treatment compensation calculator.

Simple calculators, including sliders, may be used to find a clinically acceptable compromise between offensive and defensive treatment compensation [13], an example of a smartphone based program is shown in Figure 2.

In the example in Figure 2 the *aggressivity* of treatment can be chosen based on the relative effect of the new dose per fraction on tumour as well as late effects. For typical calculations the alpha/beta ratio for tumour tissue is 10, and for normal tissue late effects: 3. The optimal compensation treatment depends on dose per fraction and number of additional treatments.

**Figure 3:**
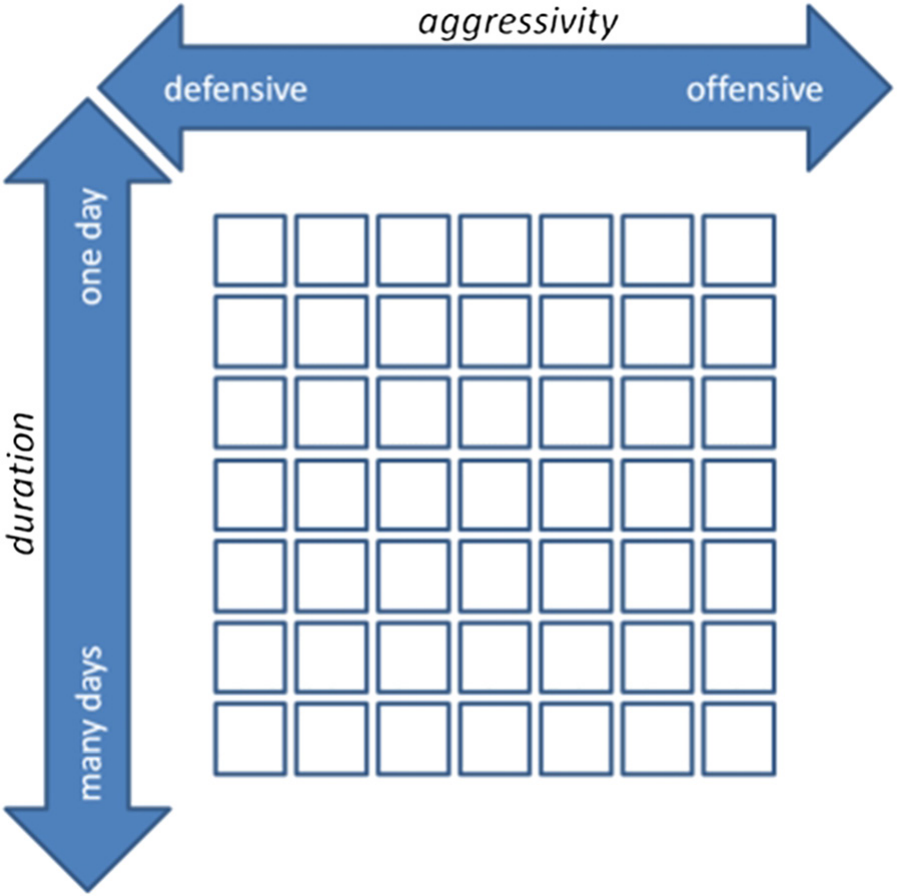
The optimal compensation treatment needs to be found on a 2 dimensional plane consisting of aggressivity and duration (days available for compensatory treatment).

The optimal compensation treatment needs to be searched for in two dimensions: *aggressivity* as well as the number of days used for compensation (Figure 3).

We developed a java based compensation treatment calculator calculating tumour as well as normal tissue doses based mostly on the calculations proposed by the Royal College of Radiologists [9].

Input into the model includes: Parameters of the planned treatment:

The planned number of fractions

The planned dose per fraction

The planned duration of treatment (the planned number of fractions + e.g. planned weekend brakes)

Parameters of the planned treatment:

The applied number of fractions

The applied dose per fraction

The duration of the applied treatment (up to the treatment brake)

Duration of the treatment brake.

Radiobiological parameters specific for tumour and normal tissue

The factor in Gy by which repopulation is accounted for (starting with day 29)

The alpha/beta value for the tumour

The alpha/beta value for late normal tissue effects

**Figure 4:**
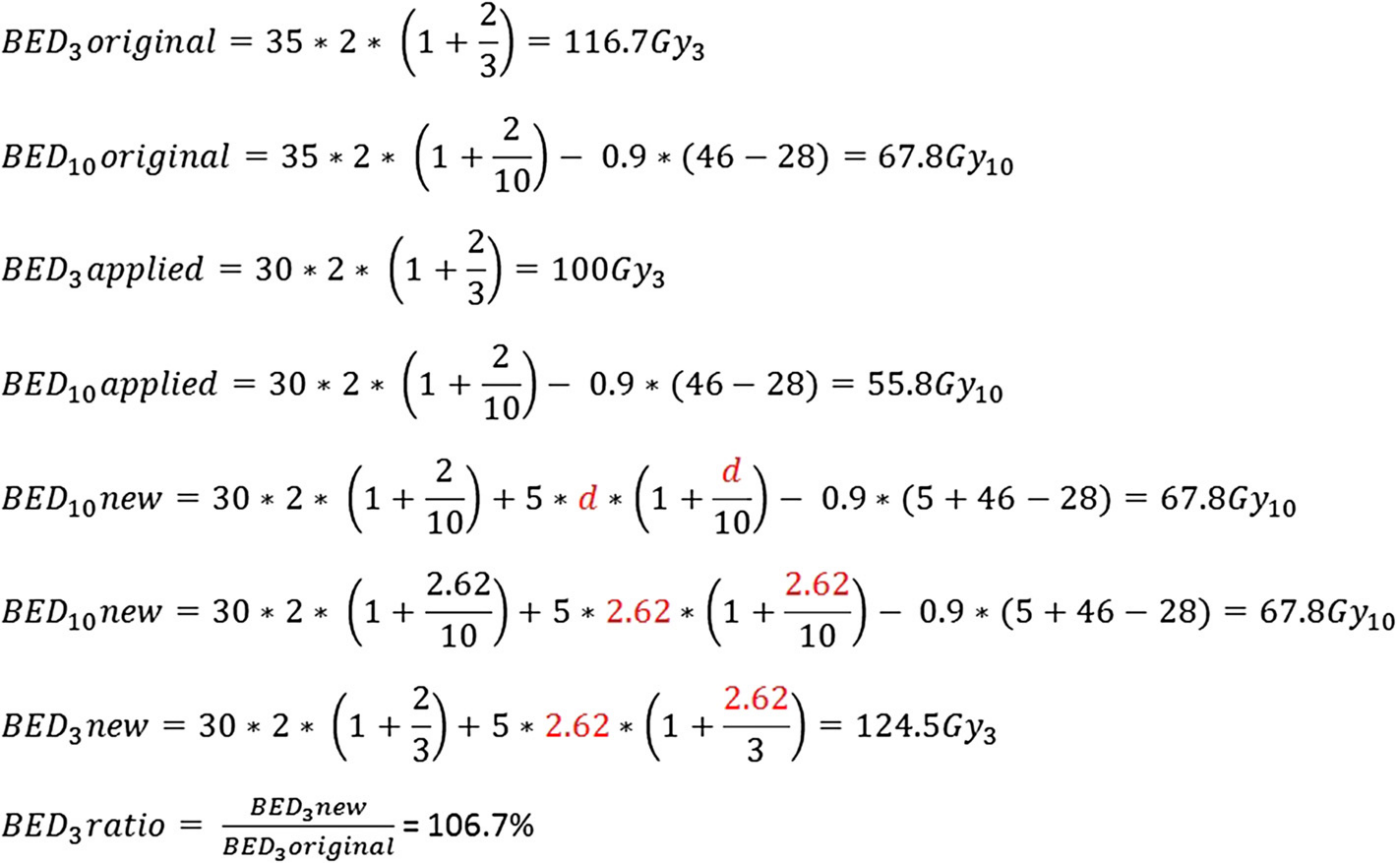
Calculating the late effect dose in percent from the compensation treatment for maximum compensation of tumour effect (offensive extreme), the tumor BED is 100% resulting in an increase of the normal tissue BED by 6.7%.

A sample calculation is shown in Figure 4. A head&neck cancer patient received only 30 of 35 planned fractions of 2Gy in the originally planned time period (46days). In this example five days are available to compensate the treatment interruption. For each day beyond the 4^th^ week of treatment, the effective tumour dose is reduced by 0.9 Gy per day. The BED_10_planned represents the biologically equivalent dose (BED) that was intended to be delivered to the tumour. The BED_3_planned represents the effect on normal tissue. The applied dose is represented by BED_10_applied and BED_3_applied respectively. To achieve the same tumour effect a total tumour BED of 67.8Gy is required (the same value as the BED_10_planned). A loss of 0.9Gy per day after the 4^th^ week is subtracted (including the 5 days of treatment prolongation due to compensatory treatment). When the formula is solved a dose per fraction of 2.62Gy is obtained. The BED_3_new is calculated using this dose per fraction and a new BED value for normal tissue is determined. The BED_3_ratio represents the ratio between BED_3_new and BED_3_original, in this case 106.7%. In this example the compensation treatment was calculated to maintain the originally planned tumour dose leading to a BED_10_ratio of 100%.

Every proposed compensation treatment results in two important values: the new tumour dose as well as the new late effects dose, each expressed in percent compared to the initially planned treatment.

In the example in Figure 4 the tumour dose would be 100%, the late effects dose would be 106.7%. Due to the nature of the sigmoid shapes of the dose response curves larger deviations from the planned treatment will probably result in over-proportional deviations in results.

We assumed it would be logical to search for a compromise between offensive and defensive treatments where both values are close to 100%. The goal should be to keep the sum of the squares of the deviations as low as possible. This value we termed the *compensability index:*

$$\begin{array}{c} \mathrm{Compensability}\;\mathrm{Index}={\left(1-\frac{\mathrm{new}\;\mathrm{tumor}\;\mathrm{effect}}{\mathrm{planned}\;\mathrm{tumor}\;\mathrm{effect}}\right)}^{2}\\ \;+{\left(\frac{\mathrm{new}\;\mathrm{normal}\;\mathrm{tissue}\;\mathrm{effect}}{\mathrm{planned}\;\mathrm{normal}\;\mathrm{tissue}\;\mathrm{effect}}-1\right)}^{2} \end{array}$$

When the treatment can be compensated for with the same tumour and late effect the compensability index is 0, with increasing deviations the compensability index increases with the sum of square powers of the deviations.

The square power was chosen for weighting the deviations as the effect would be expected to be exponential. This too is a simplification as the change in effect is also dependent on the position within the sigmoid curve. However as these details are not available for individual patients we believe the square power represents a useful practical solution.

## Results

**Figure 5:**
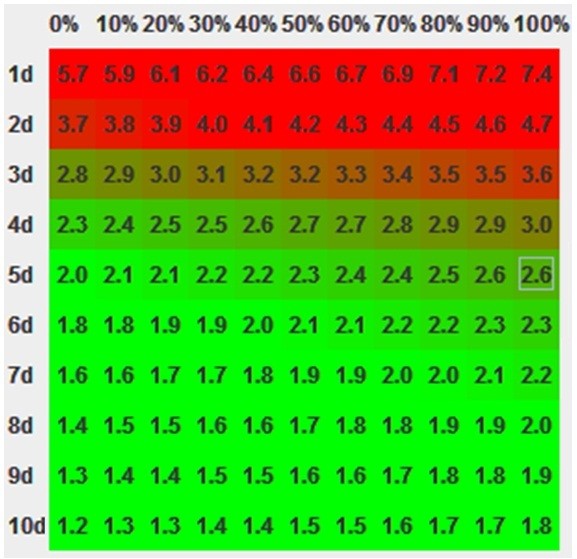
**The calculated compensation doses for the given head&neck cancer example are displayed.** The y axis represents the number of days used for compensation, the x axis represents the level of aggressivity (from defensive on the left to offensive on the right).

An automated java tool has shown to be a very efficient method of displaying all compensation options in a two-dimensional plane. Figure 5 shows the calculated doses for the given head&neck example.

The doses per fraction are displayed in green when they are the same or less, they turn to red as the doses per fraction increase.

**Figure 6:**
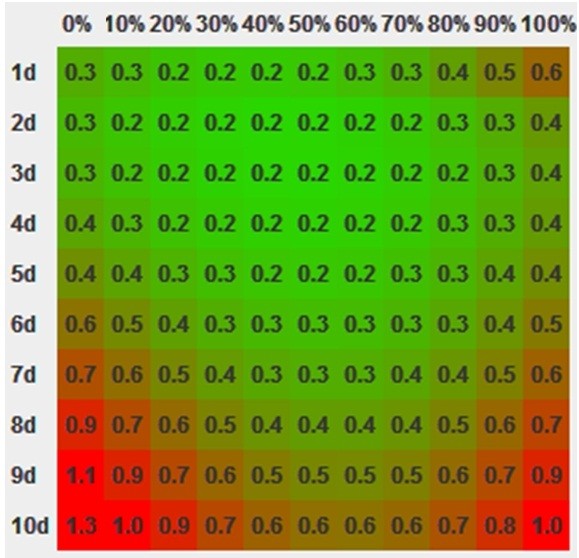
The compensability index displayed on a plane with the y axis representing the number of days for compensation and the x axis the aggressivity.

The compensability index can also be displayed on the same matrix (Figure 6).

The matrix displayed in Figure 6 provides an overview of the resulting compensability indices for various combinations of aggressivity and duration. On the horizontal axis the aggressivity ranges from 0% on the left to 100% on the right. The vertical axis represents the duration of the compensatory treatment with 1 day on the top and 10 days on the bottom. For each combination the compensatory index is calculated and displayed. Lower compensability indices (suitable) are displayed in green, the higher (less suitable) are displayed in red. The gradual change in colour also demonstrates that there are several solutions to the problem with similar results.

## Discussion

The compensability index provides a visualization method for a very complex set of information. It allows for an overview for many compensation variations (e.g. 100) at one glance. Foremost, the compensability index may help in quick orientation quiding the physician to the optimal combination of compensatory treatment days and dose per fraction.

The presented compensability index is based on a rather basic model. Concurrent chemotherapy, alternate fractionations, twice or thrice daily radiation and existing side effects are not considered currently in this concept. Additionally, the tolerance of several organs may be limiting for treatment and cannot be ignored. Other modalities such as pulse-, high- or low dose rate brachytherapy, single fraction radiotherapy or combinations of modalities are not dealt with. We believe, despite these limitations, that the compensability index may be expanded to include these topics in the future.

Depending on the clinical details; deviations from the planned tumour or normal tissue effects can be accepted without compensation. The basis of these calculations are assumed alpha/beta values, the result of the presented calculations is only as reliable as the alpha/beta estimates of normal and tumour tissues and the alpha/beta model itself.

The described calculations also do not account for individual patient characteristics, previous treatments, concurrent chemotherapy and details of dose distribution. As the compensability index does not account for the specifics of a treatment or the patient, the clinician will always have to decide on a case-to-case basis.

Any change from an established regime may be associated with increased risks, this deviation needs to be justified and the risks considered. The compensability index is not a justification for any clinical decision per se, but may serve as a decision support system.

**Figure 7:**
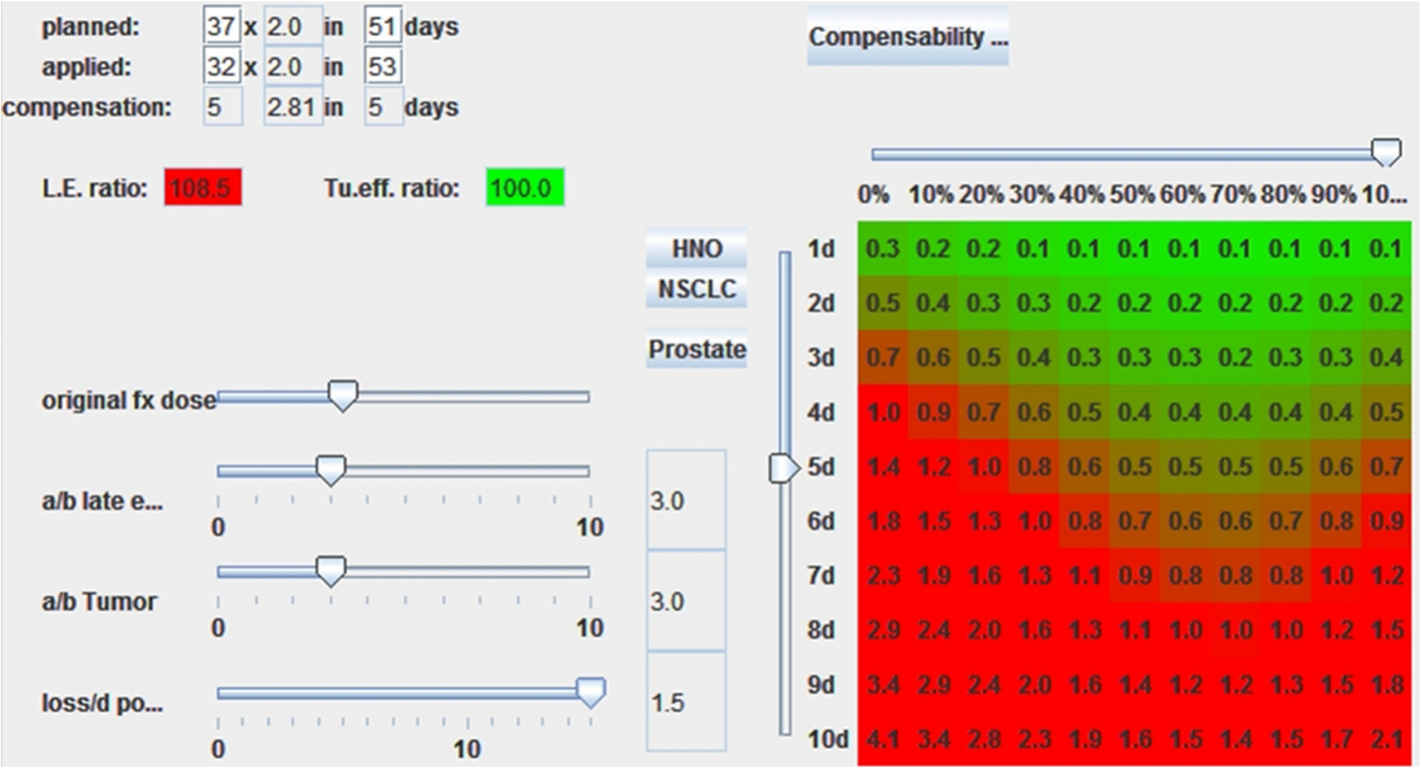
Sample interface of a compensability index calculator.

With all the known limitations the concept provides a method to quickly recalculate values on which most of our treatments are based. Graphical representation of the results also has an educational character as the effect of input parameters can be easily visualized (Figures 5– 7).

## Conclusions

The compensability index provides an efficient way to recalculate the tumour and normal tissue effects based on established and recommended formulas. A calculator-tool can be used as a decision support system, but does not replace clinical judgement. Before changing any treatment schedule a review of the treatment plan of each individual patient is mandatory.

The authors would welcome any collaboration on extending the compensability index and are happy to provide the current version for testing upon request.

## Competing interests

The authors declare that they have no competing interests.

## Authors’ contributions

PMP developed the concept and programmed the sample tool. LP provided guidance. MS and DE provided testing and radio-biological input. All authors were involved in the creation of the manuscript.
